# Maximally Informative “Stimulus Energies” in the Analysis of Neural Responses to Natural Signals

**DOI:** 10.1371/journal.pone.0071959

**Published:** 2013-11-08

**Authors:** Kanaka Rajan, William Bialek

**Affiliations:** Joseph Henry Laboratories of Physics and Lewis–Sigler Institute for Integrative Genomics, Princeton University, Princeton, New Jersey, United States of America; Harvard University, United States of America

## Abstract

The concept of feature selectivity in sensory signal processing can be formalized as dimensionality reduction: in a stimulus space of very high dimensions, neurons respond only to variations within some smaller, relevant subspace. But if neural responses exhibit invariances, then the relevant subspace typically cannot be reached by a Euclidean projection of the original stimulus. We argue that, in several cases, we can make progress by appealing to the simplest nonlinear construction, identifying the relevant variables as quadratic forms, or “stimulus energies.” Natural examples include non–phase–locked cells in the auditory system, complex cells in the visual cortex, and motion–sensitive neurons in the visual system. Generalizing the idea of maximally informative dimensions, we show that one can search for kernels of the relevant quadratic forms by maximizing the mutual information between the stimulus energy and the arrival times of action potentials. Simple implementations of this idea successfully recover the underlying properties of model neurons even when the number of parameters in the kernel is comparable to the number of action potentials and stimuli are completely natural. We explore several generalizations that allow us to incorporate plausible structure into the kernel and thereby restrict the number of parameters. We hope that this approach will add significantly to the set of tools available for the analysis of neural responses to complex, naturalistic stimuli.

## Introduction

A central concept in neuroscience is feature selectivity: as our senses are bombarded by complex, dynamic inputs, individual neurons respond to specific, identifiable components of these data [1], [2]. Neurons early in a processing pathway are thought to be sensitive to simpler features [3], [4], and one can think of subsequent stages of processing as computing conjunctions of these features, so that neurons later in the pathway respond to more complex structures in the sensory world [5]. A major challenge for theory is to make this intuition mathematically precise, and to use such a precise formulation to build tools that allow us to analyze real neurons as they respond to naturalistic inputs. There is a long history of such work, but much of it rests on the identification of “features” with filters or templates. Filtering is a linear operation, and matching to a template can be thought of as a cascade of linear and nonlinear steps. As we will see, however, there are many examples of neural feature selectivity, well known from experiments on visual and auditory systems in many organisms, for which such a description in linear terms does not lead to much simplification.

In this paper we use examples to motivate the simplest nonlinear definition of a feature, in which the relevant variable is a quadratic form in stimulus space. Because the resulting variable is connected to the “energy in frequency bands” for auditory signals, we refer to these quadratic forms as “stimulus energies.” To be useful, we have to be able to identify these structures in experiments where neurons are driven by complex, naturalistic inputs. We show that, generalizing the idea of maximally informative dimensions [6], we can find the maximally informative stimulus energies using methods that don't require special assumptions about the structure of the input stimulus ensemble. We illustrate these ideas on model neurons, and explore the amount of data that will be needed to use these methods in the analysis of real neurons.

### Motivation

To motivate the problems that we address, let us start by thinking about an example from the auditory system. This starting point is faithful to the history of our subject, since modern approaches for estimating receptive fields and filters have their origins in the classic work of de Boer and coworkers on the “reverse correlation” method [7], which was aimed at separating the filtering of acoustic signals by the inner ear from the nonlinearities of spike generation in primary auditory neurons. We will see that mathematically identical problems arise in thinking about complex cells in visual cortex, motion sensitive neurons throughout the visual pathway, and presumably in other problems as well.

We begin with the simplest model of an auditory neuron. If the sound pressure as a function of time is 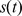, it is plausible that the activity of a neuron is controlled by some filtered version of this stimulus, so that the probability per unit time of generating a spike is
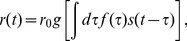
(1)where 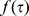 is the relevant temporal filter and 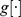 is a nonlinearity; the spikes occur at times 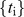. The statement that neurons are tuned is that if we look at the filter in Fourier space,
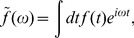
(2)then the magnitude of the filter response, 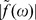, has a relatively sharp peak near some characteristic frequency 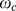. If we choose the stimulus waveforms from a Gaussian white noise ensemble, then the key result of reverse correlation is that if we compute the average stimulus in the prior to a spike, we will recover the underlying filter, independent of the nonlinearity,
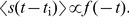
(3)We emphasize that this is a theorem, not a heuristic data analysis method [13]–[15]. If the conditions of the theorem are not met, then the spike–triggered average stimulus may provide a biased estimate of the filter. *If* the conditions of the theorem are met, then this analysis is guaranteed to give the right answer in the limit of large amounts of data.

As an aside, we note that the spike–triggered averaging method does not make assumptions about the statistics of the spike train itself. The theorem states that *if* the probability per unit time of generating single spikes can be written as in Eq. (1), and if stimuli are chosen from a Gaussian white noise ensemble, then spike–triggered averaging recovers the underlying filter. It might be that the probability of spiking is influenced by the time since previous spikes, but even if this is true there is, at each moment in time, a rate 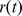 that defines the probability of spiking knowing only the history of the stimulus, not the history of spiking. It is this stimulus–dependent probability that we are writing in Eq. (1).

Equation (1) is an example of dimensionality reduction. In principle, the neuron's response at time 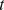 can be determined by the entire history of the stimulus for times 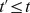. Let us suppose that we sample (and generate) the stimulus in discrete time steps spaced by 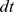. Then the stimulus history is a list of numbers
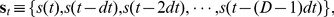
(4)where 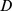 is the effective stimulus dimensionality, set by 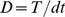, and 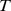 the longest plausible estimate of the integration time for the neural response. We can think of 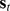 as a 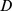
–dimensional vector. If we know that the neural response is controlled by a linearly filtered version of the sound pressure stimulus, even followed by an arbitrary nonlinearity, then only one direction in this 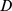
–dimensional space matters for the neuron. Further this really is a “direction,” since we can write the response as the Euclidean projection of 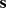 onto one axis, or equivalently the dot product between 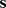 and a vector 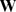,
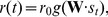
(5)where
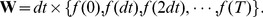
(6)This explicit formulation in terms of dimensionality reduction suggests a natural generalization in which several dimensions, rather than just one, are relevant,
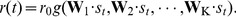
(7)As long as we have 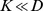, it still holds true that the neuron responds only to some limited set of stimulus dimensions, but this number is not as small as in the simplest model of a single filter.

Notice that if an auditory neuron responds according to Eq. (1), then it will exhibit “phase locking” to periodic stimuli. Specifically, if 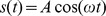 and 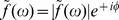, then 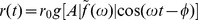. So long as there is a nonzero response to the stimulus, this response will be modulated at the stimulus frequency 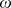, and more generally if we plot the spiking probability versus time measured by the phase 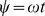 of the stimulus oscillation, then the probability will vary with, or “lock” to this phase.

While almost all auditory neurons are tuned, not all exhibit phase locking. We often summarize the behavior of tuned, non–phase–locked neurons by saying that they respond to the power in a given bandwidth or to the envelope of the signal at the output of a filter. The simplest model for such behavior, which has its roots in our understanding of hair cell responses [8]–[11], is to imagine that the output of a linear filter passes through a weak nonlinearity, then another filter. The second stage of filtering is low-pass, and will strongly attenuate any signals at or near the characteristic frequency 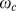. Then, to lowest order, the neuron's response depends on
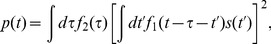
(8)where 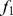 is the bandpass filter that determines the tuning of the neuron and 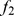 is a smoothing filter which ensures that the cell responds only to the slow variations in the power in its preferred frequency band. The probability of spiking depends on this power 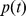 through a nonlinearity, as before,
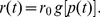
(9)Intuitively, this simple model for a non–phase–locked neuron also represents a substantial reduction in dimensionality – all that matters is the power passing through a given frequency band, defined by the filter 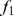. On the other hand, we cannot collapse this model into the one dimensional form of Eq. (5). To be concrete, suppose that the filter 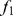 has a relatively narrow bandwidth around its characteristic frequency 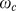. Then we can write
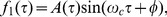
(10)where the amplitude 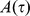 varies slowly compared to the period of the oscillation. Let us denote by 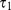 the temporal width of 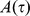, since this corresponds to the time over which the filter 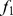 integrates the stimulus, and similarly 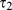 will denote the temporal width of 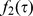. To make sure that the power 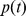 does not oscillate at (twice) the frequency 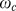, we must have 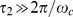. But we still have two possibilities, (a) 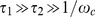 and (b) 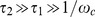. If (a) is true and there is adequate separation in the two time scales, we can show that 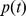 is the Pythagorean sum of the outputs of two filters that form a quadrature pair,
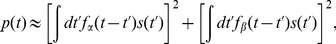
(11)where
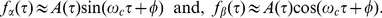
(12)On the other hand, if (b) is true, there is no simple decomposition, and the minimum number of dimensions that we need to describe this model is 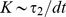, which can be quite large.

**Figure 1:**
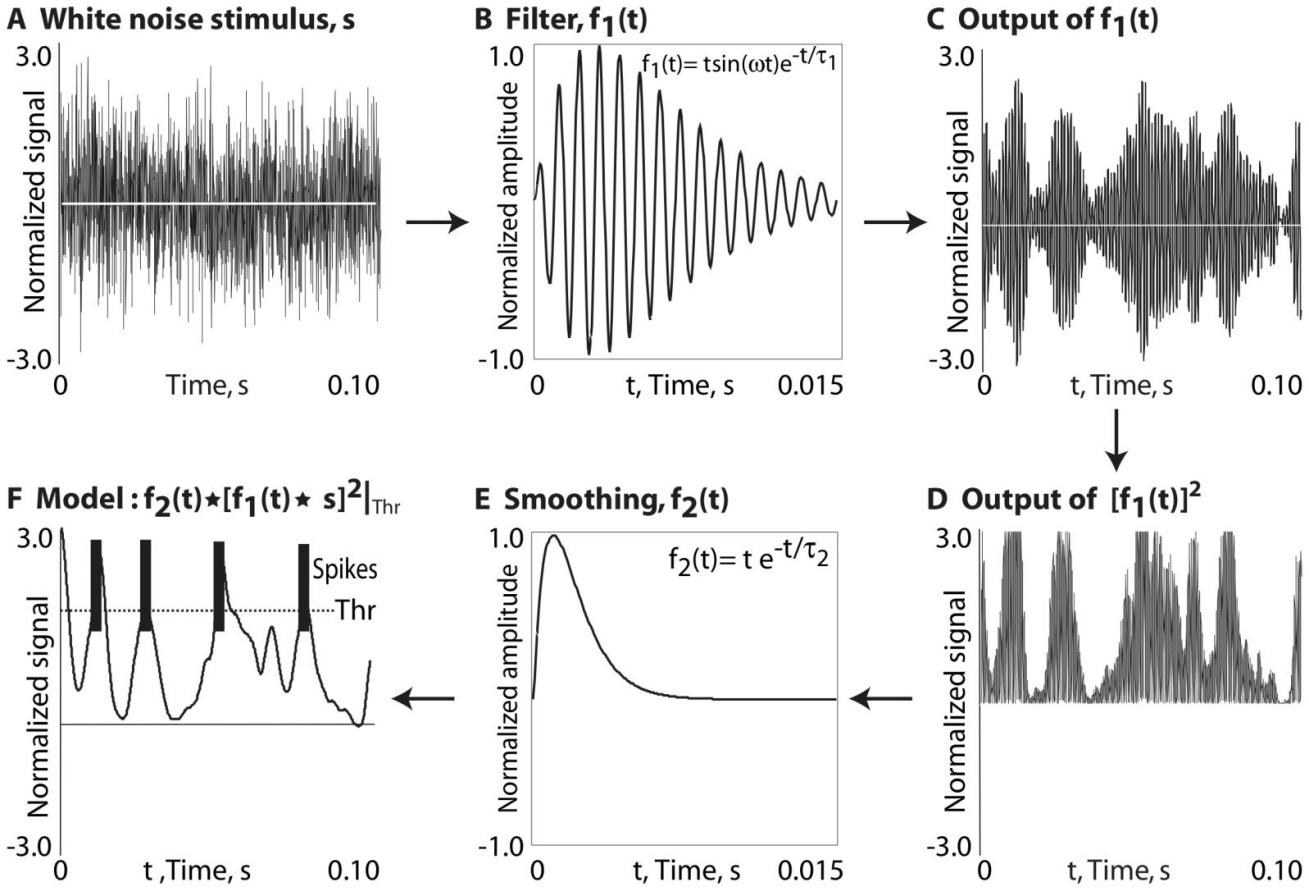
Schematic of a non–phase–locked auditory neuron. (**a**) In this implementation a model auditory neuron responds to a white noise stimulus. (**b**) The stimulus 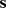 is filtered through a temporal filter 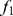, which has the form 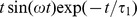 where 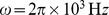 and 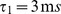. (**c**) The output of 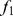 is shown here. The filter 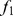 is narrow band, therefore the output oscillates at the characteristic frequency even when the input is white. (**d**) The output of 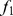 is first squared, and in (**e**), convolved with a second filter 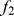 of the form 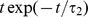, with a smoothing time constant 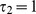 ms. (**f**) The normalized signal is finally thresholded to generate spikes. We assume that time runs discretely in steps of 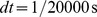.

**Figure 2:**
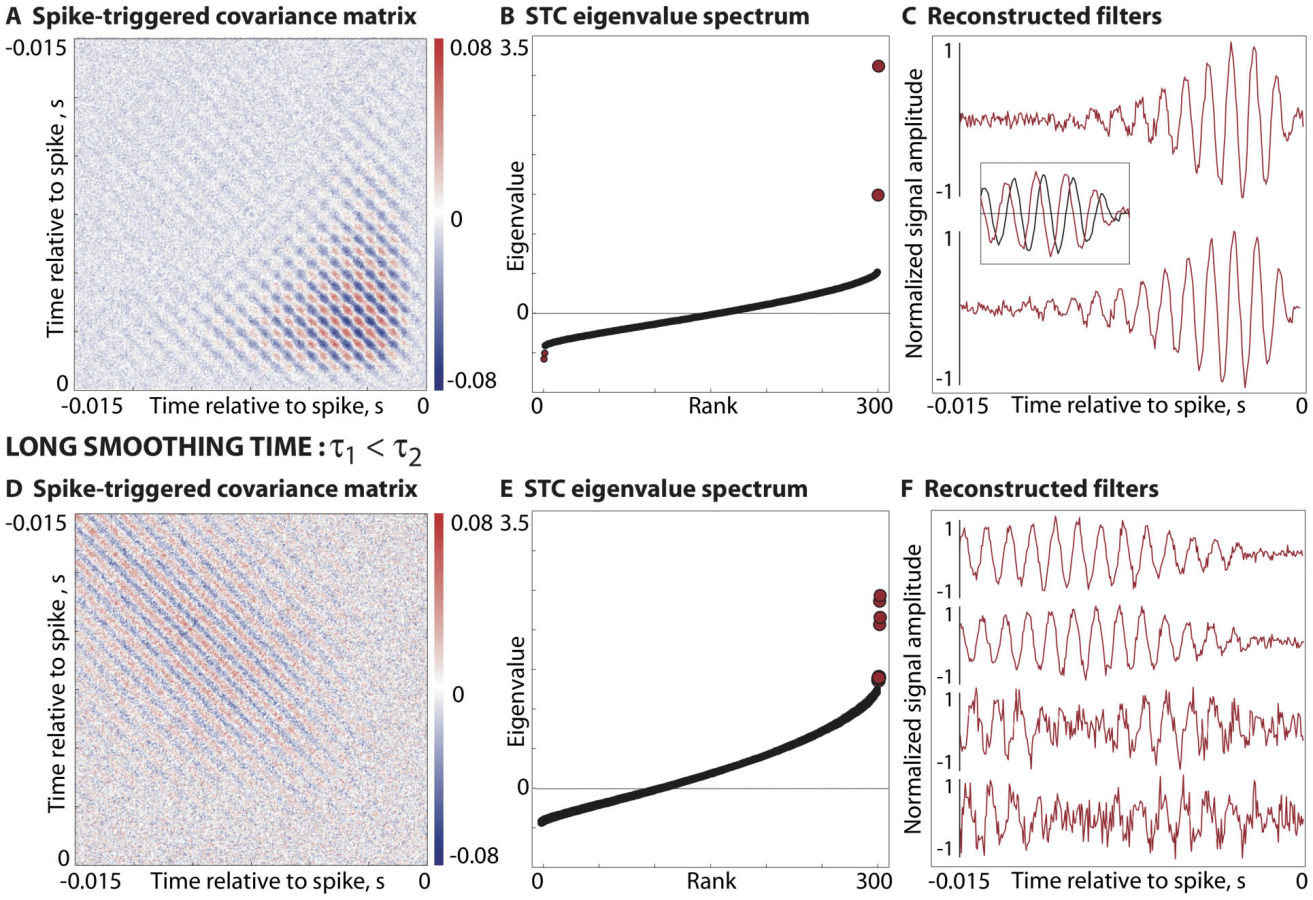
Covariance analysis of the non–phase–locked auditory neuron. (**a**) If the time constant of the smoothing filter 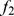 is much shorter than that of the filter 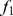, the spike–triggered covariance matrix has a relatively simple structure. Here, 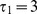
ms and 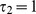
ms. (**b**) Diagonalizing this covariance matrix yields 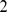 leading eigenvalues, enlarged for clarity (the rest remain close to 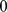). (**c**) The eigenvectors corresponding to the 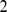 non-zero eigenvalues are the reconstructed filters plotted here. These form a quadrature pair, as shown in the inset. (**d**) If the smoothing time of the filter 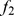 is larger than that of the first, the covariance matrix has a much richer structure. Here, 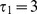
ms and 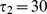
ms. (**e**) The spike–triggered covariance matrix decomposes into multiple non-unique eigenvalues, enlarged for clarity. (**f**) The eigenvectors corresponding to the non-zero eigenvalues give multiple time-shifted copies of the same filter.

We can measure the number of relevant dimensions using the spike–triggered covariance matrix. Specifically, if the stimulus vector 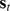 has components 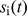, then we can form the matrix
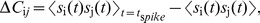
(13)where in the first term we average over the arrival time of the spikes and in the second term we average over all time. If the spiking probability behaves as in Eq. (7), and we choose the stimulus from a Gaussian ensemble, then 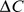 has 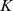 nonzero eigenvalues [12], [13], [16]–[18], [29]. In Fig. 1 we schematize the model auditory neuron we have been describing, and in Fig. 2 we show the spike–triggered covariance analysis for models in the two limits, 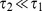 and 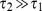. Indeed we find that in the first case there are approximately two relevant dimensions, a quadrature pair of filters, whereas in the second case there are many relevant dimensions; these dimensions appear as temporally shifted and orthogonalized copies of the filter 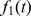.

We can think of a neuron that does not phase lock as having an invariance: it responds to acoustic waveforms that have energy in a relevant bandwidth near 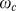, but it doesn't discriminate among signals that are shifted by small times. This invariance means that the cell is not just sensitive to one dimension of the stimulus, but to many, although these different dimensions correspond, in effect, to the same stimulus feature occurring at different times relative to the spike. Thus, we have a conflict between the notion of a “single feature” and the mathematical description of a “single dimension” via linear projection. The challenge is to provide a mathematical formulation that better captures our intuition. Before presenting a possible solution, let's see how the same problem arises in other cases.

Since the classical work of Hubel and Wiesel [19], [20], cells in the primary visual cortex are sometimes classified as simple and complex (but see [46]). Although Hubel and Wiesel did not give a mathematical description of their data, in subsequent work, simple cells often have been described in the same way that we described the simplest auditory neuron in Eq. (1) [24], [25]. If the light intensity falling on the retina varies in space (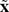) and time (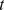) as 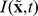, we can define a spatiotemporal receptive field 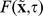 and approximate the probability that a simple cell generates a spike per unit time as
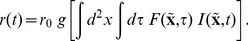
(14)If, as before, we assume that the stimulus is generated in discrete time steps (movie frames) with spacing 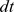, and that the stimulus influences spikes only within some time window of duration 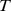, then we can think of the stimulus at any moment in time as being the 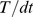 frames of the movie preceding that moment,
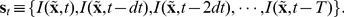
(15)If the relevant region of space is within 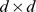 pixels, then this stimulus vector lives in a space of dimension 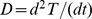, which can be enormous. As in the discussion above, Eq. (14) is a restatement of the hypothesis that only one direction in this space is relevant for determining the probability that the simple cell generates a spike, and “direction” is once again a Euclidean or linear projection.

For a complex cell, on the other hand, this single projection is inadequate (simple cells can also show sensitivity to more than one dimension [26]). Complex cells respond primarily to oriented edges and gratings, as do simple cells, but they have a degree of spatial invariance which means that their receptive fields cannot be mapped onto fixed zones of excitation and inhibition. Instead, they respond to patterns of light in a certain orientation within a large receptive field, regardless of precise location, or to movement in a certain direction. Corresponding to this intuition, analysis of complex cells using the spike–triggered covariance method shows that there is more than one relevant dimension [26]. As with non–phase–locked auditory neurons, what defeats the simplest version of dimensionality reduction in complex cells is the invariance of the response, in this case, invariance to small spatial displacement of the relevant, oriented stimulus feature [21]–[23].

The simplest model of a complex cell is precisely analogous to the quadrature pair of filters that emerge in the analysis of non–phase–locked auditory neurons. To be concrete, let us imagine that receptive fields are described by Gabor patches. The position 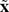 includes two orthogonal coordinates in visual space, which we call 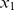 and 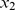. Gabor patches have an oscillatory dependence on one of these coordinates, but simply integrate along the other; both the integration and the envelope of the oscillations are described by Gaussians, so that
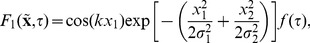
(16)and the quadrature filter is then
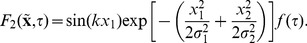
(17)Each of these filters is maximally sensitive to extended features oriented along the 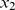 direction, but the optimal patterns of light and dark are shifted for the two filters; for simplicity we have assumed that spatial and temporal filtering is separable. If we form the energy–like quantity
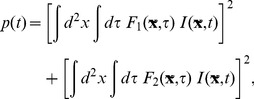
(18)we have a measure of response to oriented features independent of their precise position, and this provides a starting point for the analysis of a complex cell.

One more example of the problem we face is provided by the computation of motion in the visual system. There is a classical model for this computation, the correlator model, that grew out of the experiments by Hassenstein and Reichardt on behavioral responses to visual motion in beetles and flies [28]. Briefly, the idea behind this model is that if something is moving at velocity 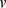, then the image intensity 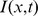 must be correlated with the intensity at 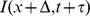, where 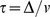. Then we can detect motion by computing this correlation and averaging over some window 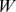 in space and 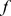 in time,
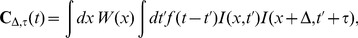
(19)where for simplicity, we think just about a single spatial dimension. In principle, with just one value of the delay 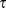 and one value of the displacement 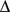, this correlation “detects” only motion at one velocity, 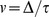, but we can easily generalize this computation to,
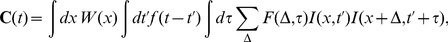
(20)where the sum over 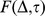 operates over different values of these spatiotemporal shifts. Depending on the precise form of this weighting we can arrange for the correlation 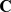 to have a relatively smooth, graded dependence on the velocity.

In the insect visual system it seems natural to think of the correlations in Eq. (20) as being computed from the outputs of individual photoreceptors, which are typically spaced 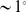 apart. In mammals, we can imagine computing a similar quantity, but we would use the outputs of larger retinal or cortical receptive fields [27]. We can also think about this computation in Fourier space. If we transform
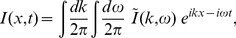
(21)then we can think of 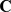 as integrating power or energy 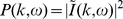 over some region in the 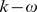 plane; motion corresponds to having this power concentrated along the line 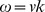. Once again there is an invariance to this computation, since as with the non–phase–locked auditory neuron we are looking for power regardless of phase. More directly, in this case the brain is trying to compute a velocity, more or less independently of the absolute position of the moving objects. Even in an insect visual system, where computing 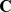 corresponds to correlating the filtered outputs of neighboring photoreceptors in the compound eye, this computation is repeated across an area containing many photoreceptors, and hence there is no way to collapse 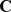 down to a function of just one or two Euclidean projections in the stimulus space.

What do these examples – non–phase–locked auditory neurons, complex cells in the visual cortex, and visual motion detectors – have in common? In all three cases, the natural, simplest starting point is a model in which the brain computes not a linear projection of the stimulus onto a receptive field, but rather a quadratic form. More precisely, if stimuli are the vectors 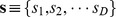, then Eq's. (8), (18) and (20) all correspond to computing a quantity
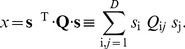
(22)Generalizing the use of terms such as “energy in a frequency band” and “motion energy,” we refer to 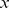 as a “stimulus energy.”

If the matrix 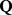 is of low rank, this means that we can build 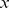 out of projections of the stimulus onto a correspondingly low dimensional Euclidean subspace, and we can try to recover the relevant structure using methods such as the spike–triggered covariance or maximally informative dimensions. Still, if 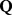 is of rank 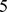 (for example), once we identify the five relevant dimensions we might run out of data before we can calculate the nonlinear input/output relation of the neuron (this problem has also been addressed in [39]) and confirm our intuition that there is indeed a single stimulus feature 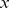 to which the cell responds.

In many cases, it is plausible that neurons could be responding just to one energy 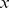, but the underlying matrix 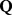 could be of full rank; a clear example is provided by correlator models for wide–field motion sensitive neurons, as in [28], [29]. But in this case there is no real “dimensionality reduction” associated with the mapping from 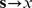, if all we know how to do is to search for linear projections or Euclidean subspaces. On the other hand, the mapping really is a tremendous reduction in complexity, because the full stimulus 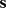 is described by 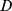 parameters, while 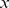 is just one number.

Suppose that the response of a neuron to complex stimuli can be described by saying that the probability of spiking depends solely on a single stimulus energy 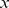 as in Eq. (22), so that
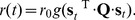
(23)Our task becomes one of showing that we can recover the underlying matrix 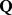 by analyzing the spike train in relation to the stimuli, without making any assumptions about the statistics of the stimuli.

## Methods

### Core of the method

If the probability of generating an action potential depends on the stimulus 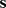, then observing the arrival of even a single spike provides information about the stimulus. Importantly, the data processing inequality [30] tells us that if we look not at the full stimulus but only at some limited or compressed description of the stimulus – a single feature, for example – we can only lose information. If the neuron really is sensitive to only one stimulus feature, however, we lose none of the available mutual information between spikes and stimuli by focusing on this one feature. This suggests a natural strategy for identifying relevant stimulus features, by searching for those which preserve the maximum amount of information [6]. Further, we can put the success of such a search on an absolute scale, by estimating more directly the information that spikes provide about the stimulus [31], [33], and asking what fraction of this information is captured by the best feature we could find.

### Setting up the problem

To make this precise, we recall that the information about the stimulus 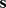 that is conveyed by the observation of a single spike can be written, following [32], as
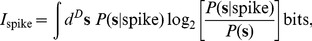
(24)where 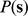 is the stimulus distribution and 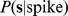 is the distribution of stimuli given that a spike occurred at a particular time (the response conditional ensemble [18]). If we consider a mapping 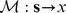, then we can also compute
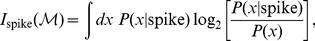
(25)knowing that for any mapping 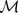, 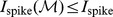. If we restrict ourselves to some class of mappings, then we can search for an 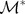 which maximizes 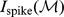, and see how close this is to the real value of 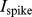, provided this can be computed from data [31]. While 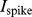 is an integral over all stimuli, and involves distributions over the full, high–dimensional, stimulus space, 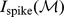 only involves distributions of a single variable 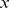. If 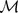 is fixed, it is relatively easy for experiments to have sufficient data that 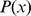 and 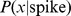 will be sampled well. Thus, as first emphasized in [6], searching for maximally informative features provides a practical and principled way to analyze neural responses to high dimensional, naturalistic stimuli.

The work in [6] considered the case where the stimulus features are one or more linear projections of the stimulus, 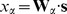, so that the mapping 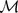 is parameterized by the vectors 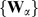; in the simplest case there being just one vector 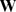. Here we are interested in quadratic mappings, corresponding to the stimulus energies in Eq. (22). Now the mapping 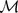 is parameterized by a symmetric matrix 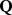. In principle, all the arguments of [6] for the linear case should hold here because the quadratic form for a full rank symmetric matrix can be rewritten as a dot product between the vectorized matrix and the vectorized product 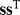. This is equivalent to the “kernel trick” in machine learning, but we note that viewing the matrix 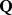 in this way, we lose track of all its structure. Before starting, we note an obvious problem related to the number of parameters we are looking for. If we are searching for a vector 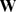 in a 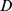
–dimensional stimulus space, we are looking an object described by 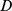 numbers. In fact, the length of the vector is irrelevant, so that maximizing 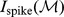 corresponds to optimization in a 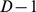 dimensional space. But if we are searching for a symmetric matrix that acts on stimulus vectors, there are 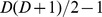 free parameters. This is a problem both because we have to optimize in a space of much higher dimensionality, and because determining more parameters reliably must require larger data sets. We will address these problems shortly, but let's start by following the path laid out in [6], which involves searching for the maximum of 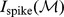 by gradient ascent.

If our stimulus feature is the energy in Eq. (22), then the distribution of 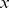 is
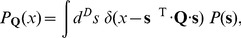
(26)where the subscript explicitly denotes that 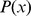 depends on 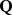. We take the derivative of this with respect to an element 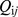 in the matrix 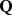,
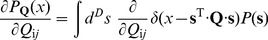
(27)

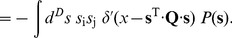
(28)Similarly, we can differentiate the distribution of 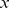 conditional on a spike,
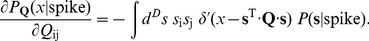
(29)Putting these terms together, we can differentiate the information:
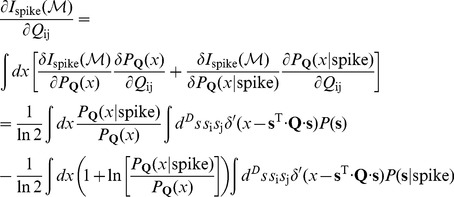
(30)

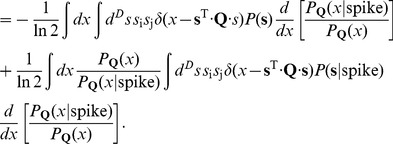
(31)But we notice that
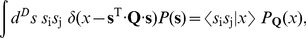
(32)where 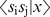 is the expectation value of 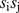 conditional on the value of the stimulus energy 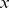, and similarly
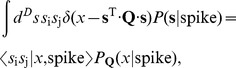
(33)where 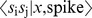 is the expectation value conditional on the energy 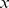 *and* the occurrence of a spike. We can combine these terms to give
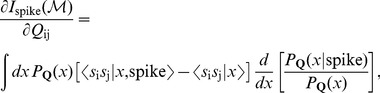
(34)or, more compactly,
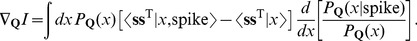
(35)To learn the maximally informative energy, or the best choice of the matrix 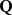, we can ascend the gradient in successive learning steps,
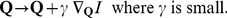
(36)


### Multiple matrices

In the same way that the idea of linear projection can be generalized to have the probability of spiking depend on multiple linear projections, we can generalize to the case where the are multiple relevant stimulus energies. Perhaps the simplest example Eq. (23) can be generalized to 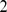 matrices, is the computation of a (regularized) ratio between two stimulus energies, such that,
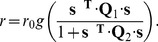
(37)Some biological examples of this formulation include gain control or normalization in V1 [26], optimal estimation theory of motion detection in visual neurons of insects [29] and complex spectrotemporal receptive fields of neurons responsible for song processing in songbirds [34], [35]. The inference task becomes one of estimating both matrices 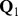 and 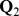 by information maximization.

As before, we can compute the gradient, noting that this time, there are two different gradients of 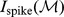,
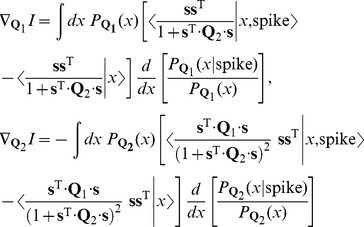
(38)Analogous to Eq. (36), at every learning step, we update each matrix 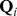 by the appropriate 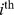 gradient,
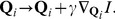
(39)where 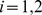 for the 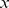 in Eq. (37). In principle the formalism in Eq. (37) could yield a more complete description of a neuron's nonlinear response properties compared to a single kernel, but there are some data-requirement challenges which we will address later.

### Technical aspects of optimization

**Figure 3:**
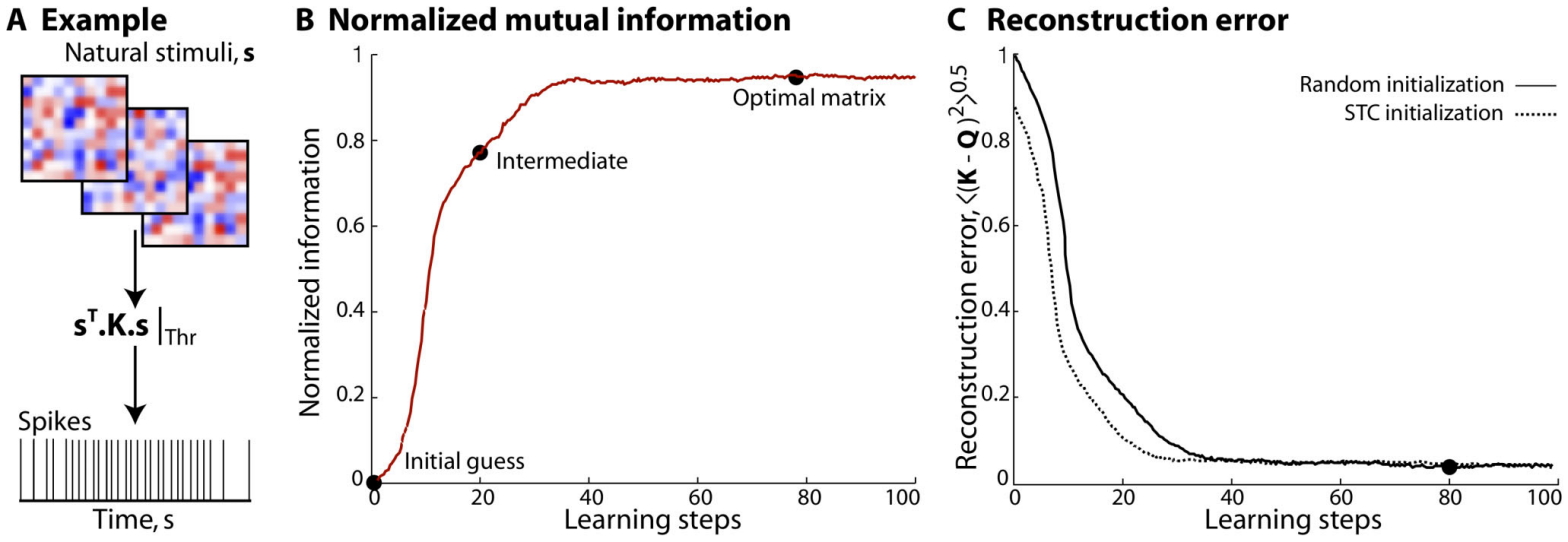
Core of the method. (**a**) A general implementation is shown here. The stimuli 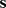 are natural image clips which are 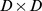 pixel patches resized from a natural image database, as described in [36]. 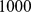 spikes are generated with a probability per time bin of 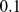 from the model neuron by a thresholding the term, 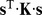 where the 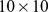 matrix 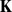 is the receptive field of the neuron. (**b**) Mutual information between the spiking response of the model neuron and the quadratic stimulus projection 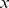 is plotted as a function of the number of learning steps. Information, normalized by its value when 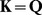, peaks at the 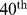 learning step and then plateaus. The 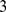 black dots on the trace denote the points at which we extract the initial, the intermediate and the optimal matrices for comparison. The maximally informative matrix 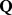 reconstructed at the 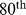 step, agrees well with 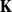, indicating convergence. For this implementation the step size 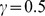 at the start and 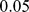 at the end of the algorithm. (**c**) Root–mean–square (RMS) reconstruction error calculated as 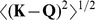, is plotted as a function of the number of learning steps. This error decreases steadily until either the randomly initialized matrix (solid line) or the matrix initialized to the spike–triggered covariance matrix (dashed line) matches 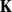. If 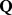 is initialized to the covariance matrix, the initial RMS error is smaller and the convergence is faster (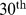 learning step) compared to that for a randomly initialized 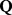. For this example, both 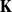 and 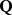 are 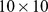 matrices and the black dot on the solid trace is at the same learning step as in panel (b).

In order to implement Eq. (36) as an algorithm, we have to evaluate all the relevant probability distributions and integrals. In practice, this means computing 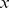 for all stimuli, choosing an appropriate binning along the 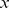
–axis, and sampling the binned versions of the spike–triggered and prior distributions. We compute the expectation values 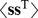 separately for each bin, approximate the integrals as sums over the bins, and derivatives as differences between neighboring bins. To deal with local extrema in the objective function, we use a large starting value of 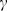 and gradually decrease 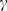 during learning. This basic prescription can be made more sophisticated, but we do not report these technical improvements here. An example of these ideas is shown in Fig. 3. We used very small patches from natural images as inputs, reshaping the intensities in nearby pixels into a 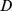
–component stimulus vector 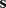 where 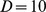. To describe the neuron we chose a random symmetric matrix 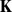 to use as the kernel, and generated spikes when the stimulus energy, 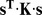, crossed a threshold, as illustrated in Fig. 3a. We fixed the spiking threshold such that the fraction of bins containing spikes is 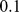, and we generated 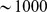 spikes. We then tried to extract the neuron's receptive field by starting with a random initial matrix 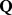, and following the gradient of mutual information, as in Eq. (36).

We let the one parameter of the algorithm, 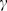, gradually decrease from a starting value of 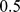 to 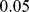, in order to minimize the fluctuations around the true maximum of the information. The relatively large initial value of 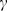 allows us to avoid simulated annealing by letting the simulation hop over false maxima. The large value of 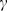 also explains why the information settles at a value slightly lower than its peak. The solution bounces back and forth from one side of this peak to the other, plateauing at a lower value than the true maximum. After this approximate solution is found, it is improved by continuing the procedure with smaller 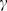 values. We normalize the resulting matrix to unit length after each gradient step.

Mutual information, the red trace in Fig. 3b, peaks at the 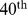 learning step and remains unchanged after that. The 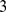 black dots in Fig. 3b correspond to the steps during the optimization when we extract and plot the initial guess, the intermediate and the optimal/maximally informative matrix 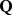. It is interesting to note that the intermediate matrix appears completely different from the optimal 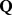 even though the corresponding mutual information is relatively close to its maximum (a similar observation was made in the context of maximally informative dimensions [6]). In general the number of steps required for the mutual information to peak and plateau depends inversely on the smoothness of the true matrix of the neuron and the amount of data available, and directly with the stimulus dimensionality. While these are only trends, a more concrete treatment of this exists in the linear case, shown in [6].

In Fig. 3c the root–mean–square (RMS) reconstruction error 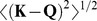 is plotted as a function of the number of learning steps for a randomly initialized 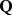 (solid line) and when 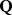 is initialized to the spike–triggered covariance (STC) matrix (dashed line). RMS error at the start of the algorithm 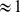 when the “true” matrix 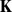 and the initial guess for 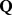 are symmetric, random matrices, uncorrelated with each other, but is slightly lower when 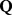 is initialized to the STC. This difference becomes smaller as the stimulus dimensionality 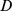 increases or as the stimulus departs more strongly from Gaussianity. Both traces decrease, and stop changing once our estimate of the optimal 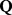 matches 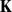. This occurs at the 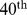 step for the randomly initialized 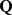 and slightly sooner (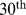 step) when initialized to the STC. If we had fewer spikes, our estimate for the optimal 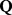 could still match 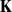 adequately, but the actual RMS error in reconstruction will be higher. We explore such performance measures and data requirement issues next.

### Performance measures and data requirements

**Figure 4:**
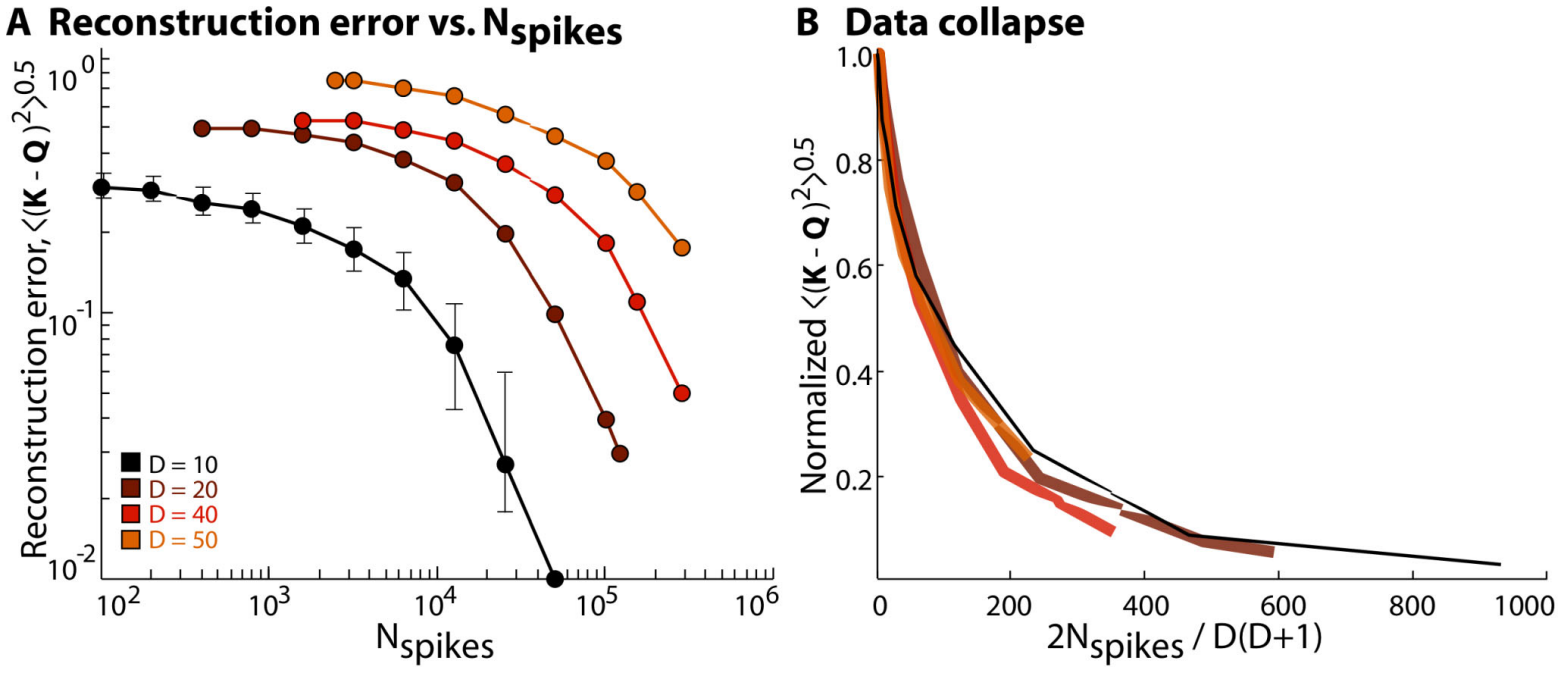
Data requirement and performance issues. (**a**) Reconstruction error 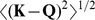 is plotted as a function of number of spikes (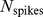) for matrices corresponding to stimuli of increasing dimensionality (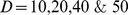). Both matrices are normalized consistently. (**b**) The traces for different values of 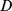 collapse when the error is normalized by the (maximum) value at 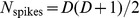 for each 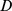, and plotted as a function of 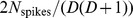.

We expect the the accuracy of our reconstruction will depend of the number of spikes 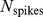. Further, we expect that when the stimulus dimensionality 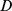 increases, the fact that there are more parameters needed to describe the kernel 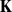 means that performance will deteriorate unless we have access to more data. To address these issues we explore model neurons as in Fig. 3, but systematically vary 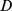 and 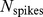. Again we consider the most difficult case, with naturalistic stimuli and kernels that are random symmetric matrices with elements drawn from a Gaussian distribution. The results are shown in Fig. 4. Our intuition is that the number of spikes should scale with the number of free parameters in the model, 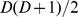, so we always start with 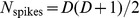. If we scale 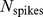 by the number of free parameters, the data collapse, as shown in Fig. 4b. Evidently accurate reconstructions of very large matrices or multiple energies requires very many spikes. This suggests that it will be important to have some more constrained models, which we now explore.

### Constrained frameworks

The most general stimulus energy is described by 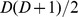 parameters, and this quickly becomes large for high dimensional stimuli. In many cases it is plausible that the matrix kernel of the stimulus energy has some simpler structure, which can be used to reduce the number of parameters.

One way to simplify the description is to use a matrix that has low rank. If, for example, the rank of the matrix 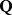 in Eq. (22) is 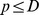, then we can find a set of orthogonal vectors 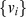 such that
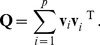
(40)In terms of these vectors, the stimulus energy is just 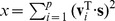.

The low rank approximation reminds us of the simpler, Euclidean notion of dimensionality reduction discussed above. Thus, we could introduce variables 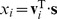 for 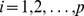. The response would then be approximated as depending on all of these variables, 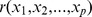, as in Eq. (7). In the stimulus energy approach, all of these multiple Euclidean projections are combined into 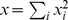, so that have a more constrained but potentially more tractable description. When 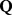 is written as 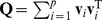, the relevant gradient of information, analogous to Eq. (35) is
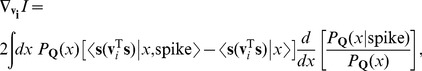
(41)and we can turn this into an algorithm for updating our estimates of the 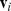,
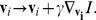
(42)There is a free direction for the overall normalization of the matrix 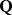 [6], [26] which makes the mutual information invariant to reparameterization of the quantities. To be sure that Eq. (40) is sufficiently general, we cannot constrain these vectors to be of unit length.

Another way of constraining the kernel of the stimulus energy is to assume that it is smooth as we move from one stimulus dimension to the next. Smooth matrices can be expanded into weighted sums of basis functions,
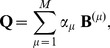
(43)and finding the optimal matrix then is equivalent to calculating the most informative 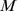
–dimensional vector of weights.

The basis can be chosen so that systematically increasing the number of basis components 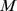 allows the reconstruction of progressively finer features in 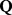. For example, we can consider 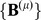 to be a set of Gaussian bumps tiling the 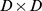 matrix 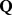, and whose scale (standard deviation) is inversely proportional to 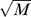. For 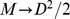 the basis matrix set becomes a complete basis, allowing every 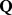 to be exactly represented by the vector of coefficients 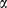. In any matrix basis representation, the learning rule becomes,
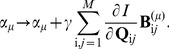
(44)This is equivalent to taking projections of our general learning rule, Eq. (36), onto the basis elements.

## Results

### A model auditory neuron

**Figure 5:**
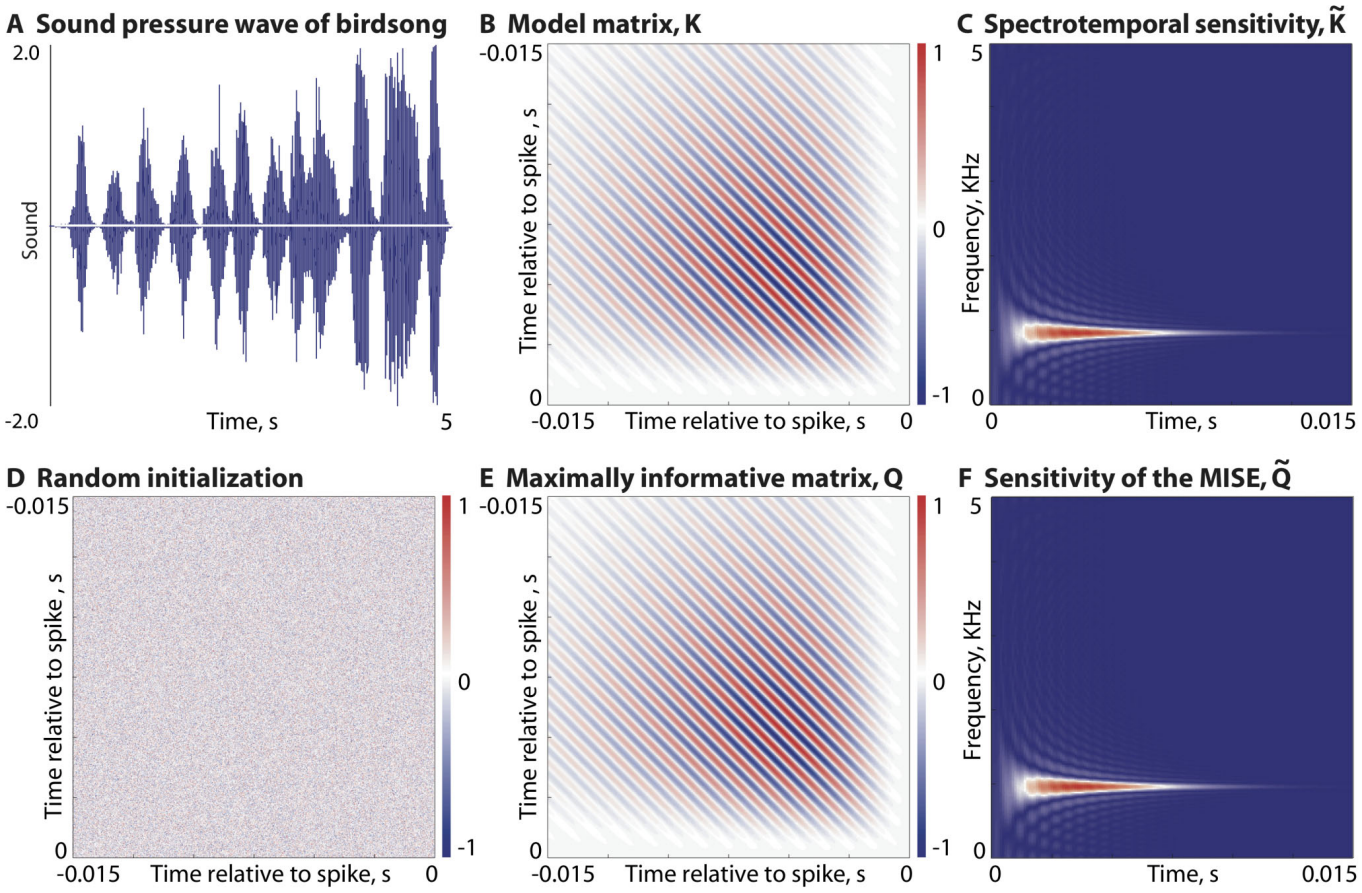
Analyzing the responses of the model auditory neuron to a bird song. (**a**) The sound pressure wave of a zebra finch song used as stimulus to the model neuron is shown here. (**b**) The equivalent matrix 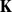, constructed from the two filters as described in Eq. (8) is 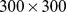 in size but has a relatively simple structure. (**c**) Taking a Fourier transform over 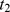 of 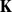 yields a spectrotemporal sensitivity matrix, 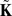 with a peak at 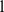
kHz. (**d**) The initial guess for 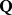 is the random symmetric matrix plotted here. (**e**) The optimal matrix 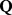 that maximizes the mutual information between the spiking response of the model neuron and the projection 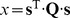 matches 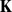 well at the end of 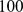 learning steps. (**f**) The spectrotemporal sensitivity 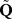, corresponding to the maximally informative stimulus energy has the same response preferences as 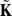 at 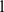
kHz.

As a first example, we return to the model auditory neuron whose response properties from Eq's (8) and (9) were schematized in Fig. 1. Rather than studying its responses to white noise stimuli, however, we consider the responses to bird song, as shown in Fig. 5a. We start with Eq. (8) and see that it is equivalent to a stimulus energy with kernel 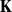 defined through
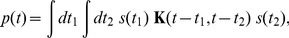
(45)

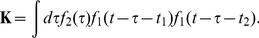
(46)We used the same filters as we showed in Fig. 1b and Fig. 1e to construct 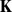, which is plotted in Fig. 5b. We can also look in a mixed time–frequency representation to generate a spectrotemporal “sensitivity,” 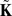, as follows:
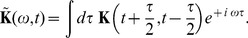
(47)

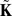
 is a characteristic of the model neuron and describes its selectivity to the stimulus. We see that this neuron responds to sound with frequencies around 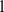
kHz with a temporal dependence dictated by the time constants of the 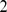 filters that make up the neuron's receptive field matrix 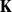, as shown in Fig. 5c. This description has the flavor of a spectrotemporal receptive field (STRF), but in the usual implementations of the STRF idea a spectrogram representation is imposed onto the stimulus, fixing the shapes of the elementary bins in the time–frequency plane and assuming that the cell responds only to stimulus power in each frequency band. Here, in contrast, Fourier transforms are in principle continuous, and the general quadratic form allows for more than just stimulus power to be relevant.

The natural stimuli we used to probe this model auditory neuron's receptive field came from recordings of zebra finch songs, modified into stimulus clips 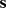. The songs were interpolated down from their original sampling rate to retain the same discrete time steps (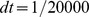 s) that we use in Fig. 1. An example sound pressure wave of a song stimulus is plotted in Fig. 5a.

This model neuron, as illustrated in Fig. 1, emitted spikes when the power in Eq. (46) exceeded a threshold. We set the threshold of firing so that the mean spike rate was 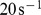. We presented 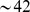 minutes of bird song stimuli to the model neuron, collecting roughly 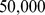 spikes. We follow the gradient ascent procedure exactly as described in Eq. (36). Note that 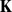 in this model is a 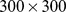 matrix, and we make no further assumptions about its structure; we start with a random initial condition (Fig. 5d). The maximally informative matrix that we find is shown in Fig. 5e, and is in agreement with the matrix 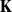; we can see this in the frequency domain as well, shown in Fig. 5f. Quantitatively, the RMS reconstruction error for this inference is less than 5% of the maximum for any two uncorrelated random, symmetric matrices of the same size.

### Model complex cells

**Figure 6:**
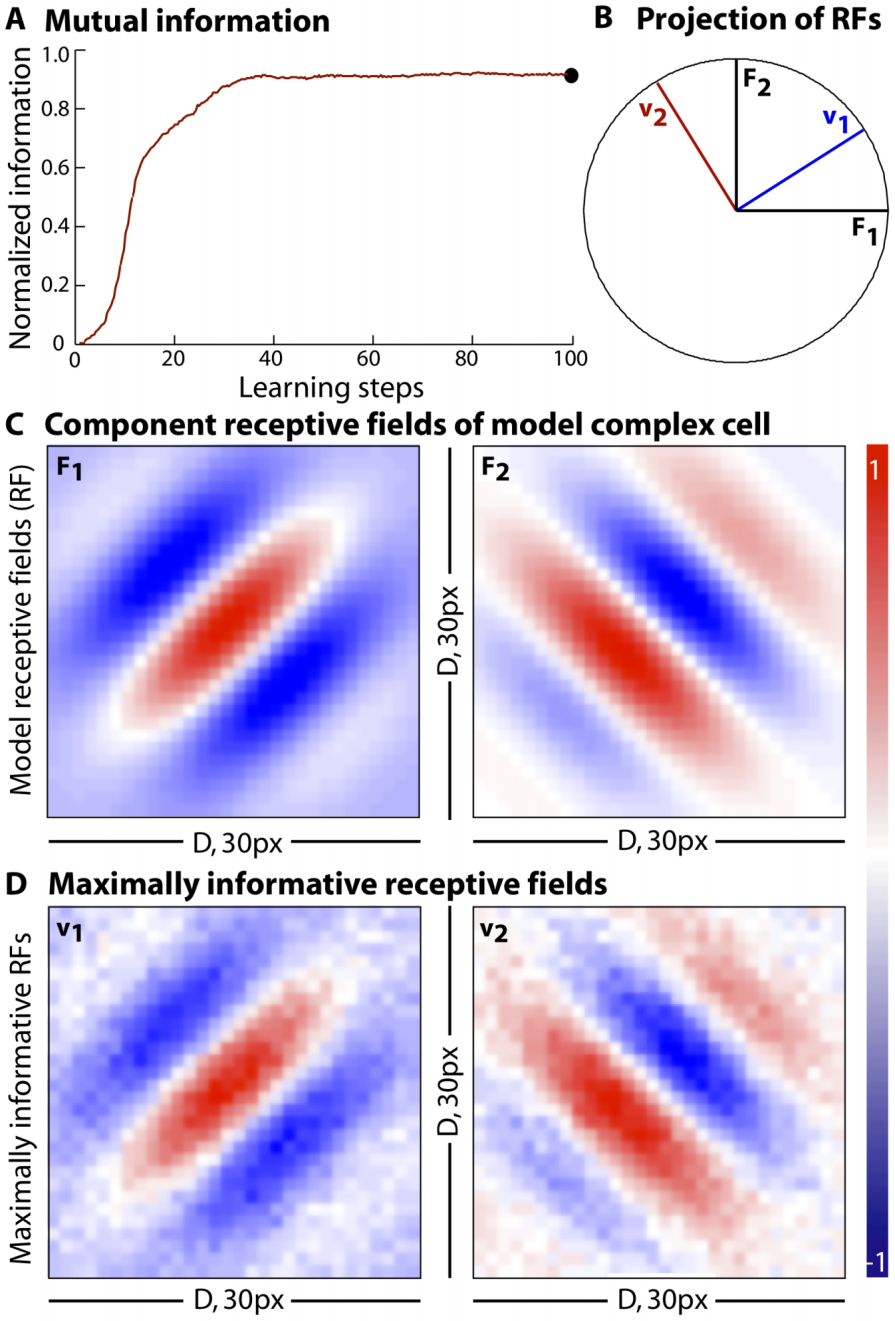
Receptive fields of a model complex cell and the reconstructed maximally informative pair. (**a**) Information as a function of the number of learning steps peaks and then plateaus. The black dot is the point where the reconstructed receptive fields are shown in panel (d) below. (**b**) Reconstructed vectors 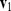, 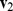 are rotated versions of the receptive fields 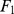, 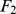, but span the same linear subspace (all vectors are normalized to unit length). (**c**) The receptive field of the model complex cell is given by the two linear filters in Eq. (18): 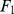 (left) and 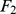 (right). (**d**) The reconstructed receptive fields at the 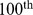 learning step (black dot in panel (a) above) with filters 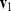 (left) and 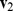 (right) rotated to best align with the 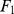 – 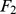 pair.

We consider the model complex cell described earlier in Eq. (18), but allow integration over just one frame of a movie, so we don't need to describe the temporal filter. We chose parameters 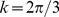, 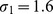 and 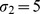, with positions measured in pixels. The stimuli were 20,000 grayscale 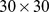 pixel image patches extracted from a calibrated natural image database [36]. Spikes were generated as before, with a threshold set to ensure that the fraction of bins containing spikes is 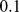. We note that, in this model, the kernel is explicitly of rank 2, and so we followed the algorithm in Eq. (41). The results are shown in Fig. 6. As expected, the best possible reconstruction is a vector pair 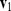, 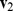 that is equal to the pair 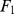, 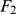 up to a rotation.

**Figure 7:**
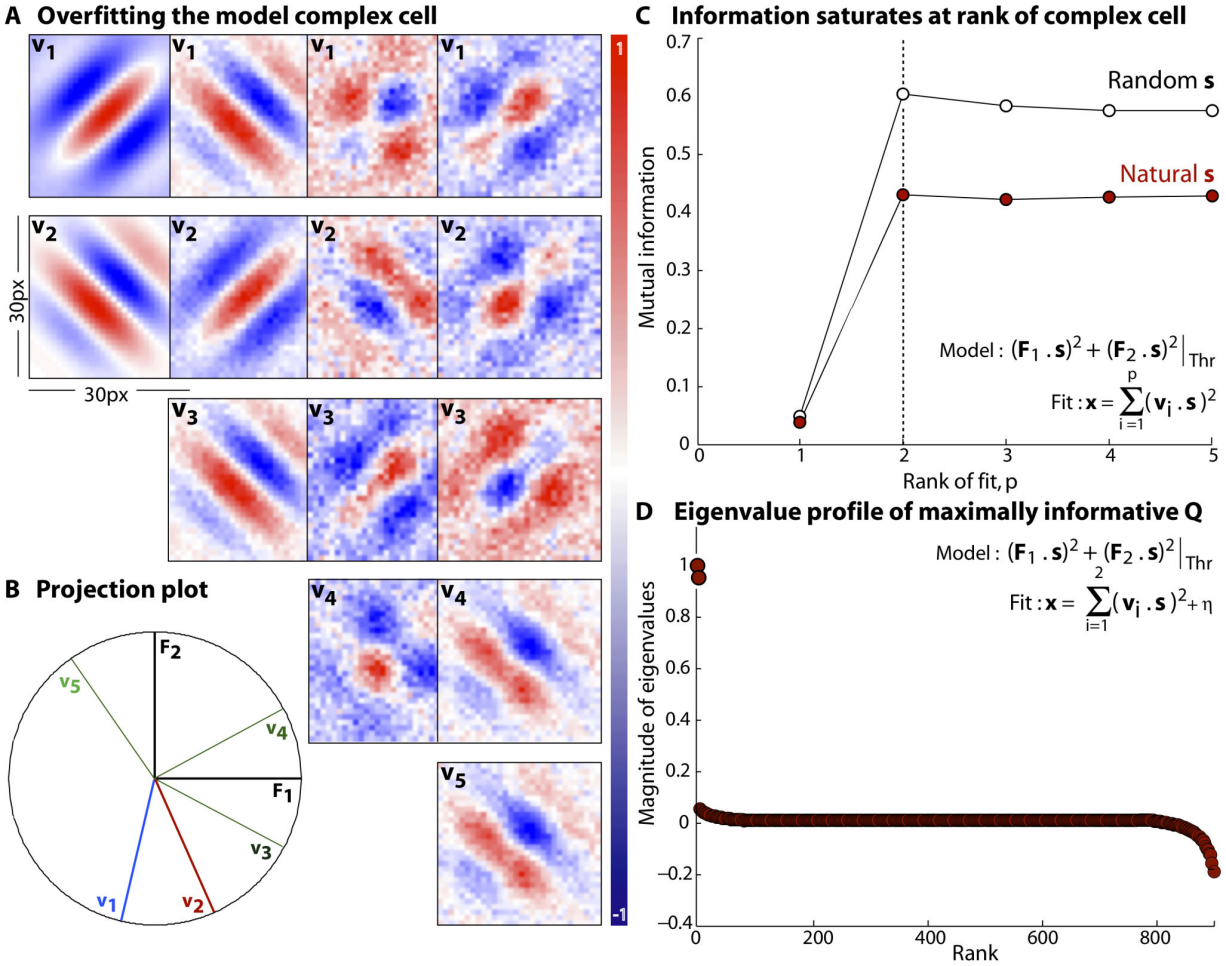
Over-fitting the model complex cell with matrices of successively increasing rank. (**a**) Receptive fields reconstructed after mutual information is maximized with matrices of rank 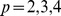 and 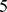 (from left to right). (**b**) The resulting vectors, 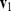 through 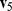, at the end of the information maximization are no longer orthogonal but project fully into a unit circle in the 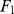
–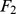 plane. (**c**) Maximum mutual information as a function of the rank of fit, 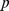, for random stimuli (open circles) or for the stimulus matrix generated using natural scenes (filled circles), peaks at the rank equal to that of the “data” (rank 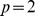 for the model complex cell), and remains unchanged as the rank of 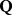 increases. (**d**) Over-fitting the model matrix 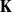 with Gaussian noise does not add to the mutual information 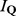 and the algorithm successfully finds a two dimensional solution. Eigenvalue profile of matrix 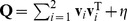 where 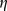 is a 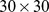 sized-random matrix after maximizing information with respect to the complex cell. Aside from two leading eigenvalues with magnitude 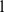, the rest 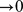.

Suppose that the real neuron, as in our model, is described by a kernel of rank 2, but we don't know this and hence search for a kernel of higher rank. As shown in Fig. 7c, higher rank fits do not increase the information that we capture, either for random stimuli or for natural stimuli. Interestingly, however, the “extra” components of our model are not driven to zero, but appear as (redundant) linear combinations of the two true underlying vectors, so that the algorithm still finds a genuinely two dimensional, albeit over–complete, solution. We could also initialize the algorithm with a full rank matrix, 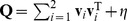, where 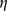 is Gaussian random noise. The convergence of 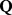 to the “true” matrix 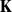 can be determined by looking at the projections of the leading eigenvectors of the matrix 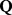 at the end of the maximization algorithm. In this complex cell example where spikes are generated from a rank 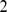 matrix, the eigenvectors corresponding to the two leading eigenvalues of the fit matrix 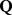 should be identical to 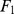, 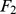. The remaining eigenvalues should be driven to be 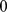, and this is indeed what we see in Fig. 7d.

### Matrix basis formalism

**Figure 8:**
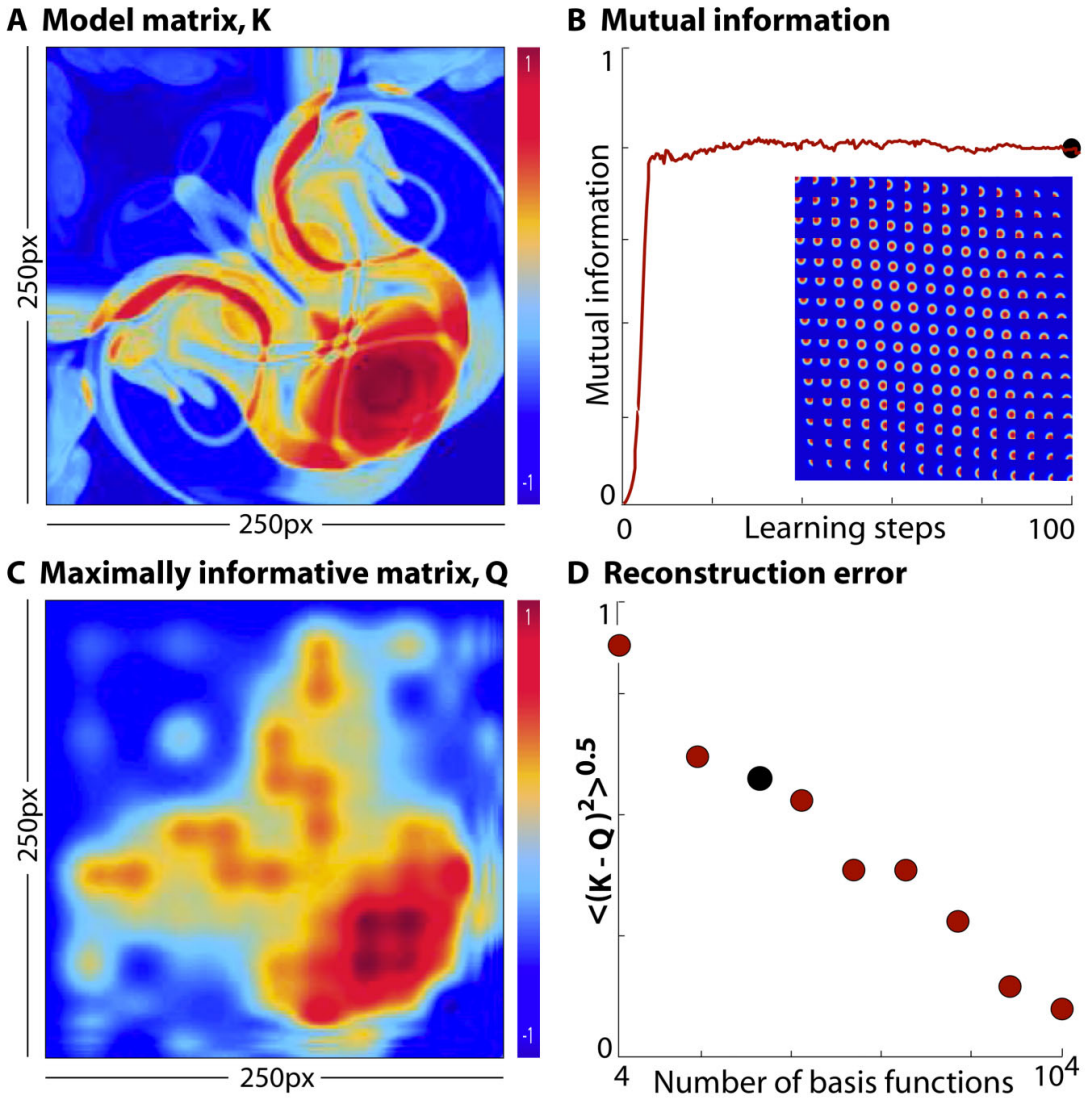
Matrix basis formalism for constraining the number of free parameters. (**a**) A complex, structured full rank matrix 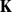 is generated by symmetrizing a 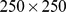 pixel image of a fluid jet, and used as the “true” kernel for our model neuron. (**b**) Mutual information increases with the number of learning steps, peaks at the 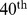 step and remains unchanged thereafter. Inset shows the collection of 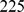 Gaussian matrix basis functions whose peaks densely tile the space of 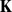. A trial matrix is constructed as a linear sum (with coefficients 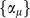) of the basis matrices, and information optimization is performed over 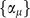. The black dot at the 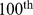 learning step is the point where 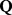 is extracted. (**c**) The reconstructed matrix kernel 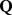 after maximizing mutual information using the 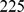 basis coefficients making up the kernel is shown here. (**d**) The RMS reconstruction error 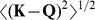 decreases as the number of basis functions 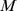 increases from 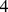 to 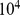. With enough data perfect reconstruction is possible as 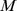 approaches the number of independent pixels in 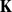.

Here we explore the matrix basis formalism, as described in Eq. (43), to infer a matrix 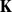 that could be of arbitrarily high rank. For 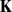 we used a symmetrized 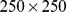 pixel image of a fluid jet as shown in Fig. 8a. While this is not an example of a receptive field from biology, it illustrates the validity of our approach even when the response has an atypical and complex dependence on the stimulus. Spikes were generated by thresholding the energy 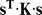, and the same naturalistic visual stimulus ensemble was used as before. Gaussian basis matrices shown in the inset of Fig. 8b were used to represent the quadratic kernel, reducing the number of free parameters from 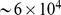 to 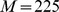. We start the gradient ascent with a large 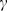 value of 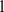 and progressively scale it down to 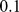 near the end of the algorithm; Fig. 8b shows the information plateauing in about 40 learning steps. The maximally informative quadratic kernel 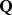 reconstructed from these 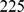 basis coefficients is shown in Fig. 8c. Optimizing the coefficients of the 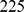 basis functions captures the overall structure of the kernel, and this can be improved to an almost perfect reconstruction (at a pixel–by–pixel resolution) by increasing 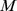, as shown in Fig. 8d. The reduction in the number of parameters to be estimated through this simplification means that this method can be easily extended to the inference of much larger matrices, with a precision dictated by the amount of data available.

### Multiple matrices

**Figure 9:**
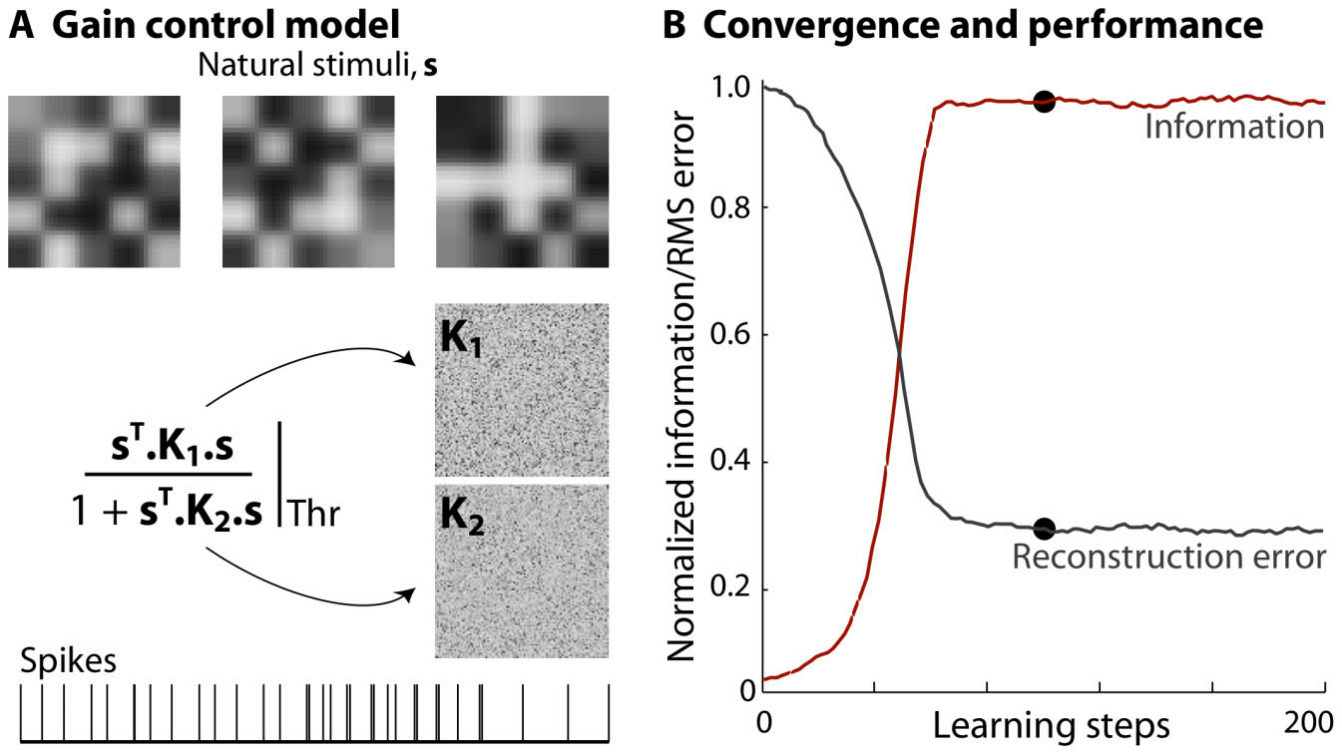
Inferring 2 maximally informative energies simultaneously. (**a**) Schematic of a model neuron with divisive gain control as described in the text. Natural images were reshaped and used as stimulus clips, 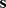. The “true” matrices 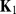 and 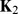 were generated as random, symmetric 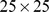 matrices. (**b**) Mutual information normalized by the maximum when 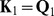 and 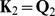, peaks at the 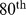 learning step and remains unchanged after. RMS reconstruction error (green), computed as 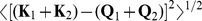 decays to a steady low at the same step. Using more spikes will decrease this further.

We consider a model neuron similar to the one we described in Eq. (37), with stimulus dimensionality 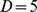; the two matrices, 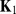 and 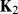, are plotted in Fig. 9a, along with a schematic of the spikes produced when the nonlinearity 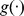 is a threshold. Again we constructed stimuli from nearby pixels of natural images, so that the distribution is strongly correlated and non–Gaussian; the spiking threshold was set so that the fraction of bins with spikes to 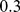, and we generated 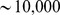 spikes. We followed the algorithm in Eq's (38) and (39) to find the maximally informative matrices 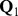 and 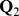. Mutual information normalized by the maximum when 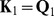 and 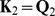, and RMS reconstruction error in green (calculated as 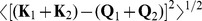) are plotted as a function of the learning steps in Fig. 9c. While convergence is definitely possible, our estimates of the maximally informative matrices are noisier than in the single matrix instances, even with a relatively large amount of data. We expect that realistic searches for multiple stimulus energies will require us to impose some simplifying structure on the underlying matrices.

## Discussion

There is a long history in neuroscience of trying to extract receptive fields from neural responses to a variety of synthetic and natural stimuli [40]. While the field began with a focus on linear models for feature selectivity, more complex, nonlinear notions of “features” have been explored as well; some of these studies go back several decades. In the Y-type retinal ganglion cells of the cat responding to multi-sinusoidal signals, for instance, the responses were fit by incorporating second order kernels [41], [42]. If we start with a model in which, as discussed here, the probability of spiking is determined by a quadratic “stimulus energy,” then the second order kernel is precisely the first term in a series expansion of the probability as a function of the energy; our approach thus connects to a large body of work using series expansion methods (e.g., Wiener and Volterra series) to characterize the input/output relations of neurons [43]. The class of models we consider allows for a more or less arbitrary nonlinear relationship between the spike probability and the stimulus energy, thus summing up a selection of all higher order terms in a series expansion.

There have also been other more recent efforts that point to the importance of quadratic forms in stimulus space. It is shown in [37] that a logistic dependence of the spike probability on a quadratic form is the maximum entropy, and hence least structured, model consistent with a measurement of the spike–triggered covariance matrix, and that this identification is correct without any assumptions about the distributions of inputs. In addition [38], compares information theoretic extensions of spike-triggered covariance to non-Gaussian stimuli and concludes that when the number of free parameters is large, the non-convex nature of multiple MID optimizations could raise issues. In a different direction, the work in [39] considers models in which the spike probability depends exponentially on a quadratic form, and spiking is explicitly a Poisson process. They show that this model, and some generalizations, lends itself to a Bayesian formulation, in which various simplifying structures (see Section IV) can be imposed through prior distributions on 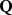. In some limits, the different recent approaches are equivalent to one another, and to the search for maximally informative stimulus energies that we propose here (see [45]).

The maximally informative stimulus energies we have developed can be generalized to include the inference of an arbitrary two-dimensional, nonlinear quantity of the form 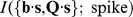 without an explicit assumption about how the linear and the quadratic part combine. This idea is explored further in [45]. In a similar spirit, searching for multiple stimulus energies (shown in Fig. 9) would allow us to discover sensitivity to different sorts of nonlinear combinations, ranging from divisive nonlinearities and normalization, to logical AND operations among multiple features, without having to assume a specific form for these interactions in advance.

An important aspect of methods based on information theoretic measures is that the quantities we compute have a meaning beyond more detailed models, and our results can be calibrated on an absolute scale. As an example, almost no neuron in the brain behaves exactly as a modulated Poisson process, in which the arrival times of successive spikes are independent given the stimulus. Nonetheless, there is a well defined question as to which stimulus features are most informative about the arrival times of individual spikes. Single spikes may acquire sensitivity to multiple stimulus features through interactions with other spikes, but finding these features, or the most informative single feature, does not require us to make assumptions about the spike statistics. Similarly, in experiments where stimuli are repeated we can estimate the information carried by the arrival times of single spikes or more complex events, without assumptions about the interactions among these events [32], [44], and this provides an absolute calibration for the analysis of feature selectivity: we can ask not just for the features that capture the maximum information, but we can compare this feature-based information to the total, and ask if a description in terms of a small number of features really works.

In conclusion, while the notion that neurons respond to multiple projections of the stimulus onto orthogonal filters is powerful, it has been difficult to develop a systematic framework to infer a neuron's response properties when there are more than two filters. To get around this limitation, we propose an alternative model in which the neural response is characterized by features that are quadratic functions of the stimulus. In other words, instead of being described by multiple linear filters, the selectivity of the neuron is described by a single quadratic kernel. The choice of a quadratic form is motived by the fact that many neural phenomena previously studied in isolation can be viewed as instances of quadratic dependences on the stimulus. We presented a method for inferring maximally informative stimulus energies based on information maximization. We make no assumptions about how the quadratic projections onto the resulting matrices map onto patterns of spiking and silence in the neuron. This approach yields asymptotically unbiased estimates for receptive fields for arbitrary ensembles of stimuli, but requires optimization in a possibly rugged information landscape. While clearly only one step toward a more complete collection of such tools, the methods we have presented should help elucidate how sensitivity to high–order statistical features of natural inputs arises in a wide range of systems.
